# Bridging bronchus - a rare cause of recurrent wheezy bronchitis

**DOI:** 10.1186/1471-2431-12-110

**Published:** 2012-07-28

**Authors:** Anja Schnabel, Katja Glutig, Christian Vogelberg

**Affiliations:** 1 Pediatric Department, Technical University of Dresden, Germany; University Hospital, Fetscherstraße 74, 01307, Dresden, Germany; 2 Department of Pediatric Radiology, Technical University of Dresden, Germany; University Hospital, Fetscherstraße 74, 01307, Dresden, Germany

**Keywords:** Congenital airway anomalies, Bronchial branching anomaly, Airway malformation

## Abstract

**Background:**

Wheezing is a common symptom in infants and toddlers. Usually it occurs due to viral infection of the lower airways and no further diagnostic procedures are necessary. However in rare cases, other reasons such as anatomical malformation have to be considered.

**Case presentation:**

We report about an infant with recurrent episodes of wheezy bronchitis, which persisted despite adequate therapy. Bronchoscopy and computed tomography of the lung with three-dimensional reconstruction revealed a rare bronchial branching anomaly - the so called “bridging bronchus”. In contrast to previous case reports, this infant showed no additional malformations, which seems to be important for the prognosis.

**Conclusion:**

To the best of our knowledge this is the first report about a patient with a bridging bronchus in its “original form” without associated anomalies of the trachea-bronchial system or other organs.

## Background

Wheezing is a common symptom in infants and toddlers with at least one episode in 45% and recurrent episodes in 20% of them respectively during their first 12–15 months of life [1]. Principally wheezing results from accelerated air flows through a narrowed segment of the respiratory tree, however, there is a wide range of differential diagnosis of wheezing in children which makes it sometimes necessary to step up with the diagnostical procedure. Most cases with intermittent wheezing are due to viral infections, especially due to *Respiratory Syncytial Virus* (RSV) [2]. Other reasons might be bronchial asthma, cystic fibrosis, foreign body aspiration or gastroesophageal reflux [3]. In case of treatment failure, rare reasons should be considered, including anatomical malformations of the bronchial tree. We report about a female toddler with recurrent episodes of wheezing due to an extremely rare bronchial branching anomaly.

## Case presentation

**Figure 1:**
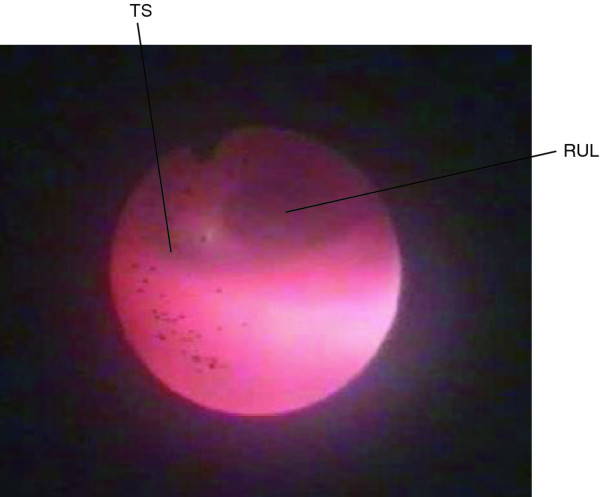
Endoscopic aspect of the tracheal stenosis (left side).

**Figure 2:**
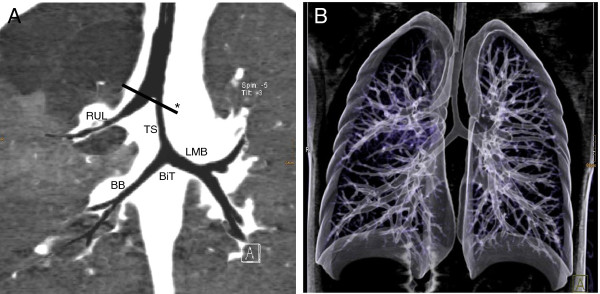
**A) 2-dimensional and B) 3-dimensional computed tomography reconstruction of the tracheo-bronchial system.** Bridging bronchus (BB) originating in the left main-stem bronchus (LMB) crossed the mediastinum and provided the right lower and middle lobe. Trachea (T), right upper lobe bronchus (RUL), bifurcatio tracheae (BiT), tracheal stenosis (TS). Appropriate view of Figure 1 (*).

A 2630 g female newborn was delivered at term by a cesarean section due to deceleration after an uncomplicated pregnancy to a 36-year-old mother. Since the age of three months she was hospitalized several times with intermittent wheezing, inspiratory stridor, epigastrical retractions, dyspnea and cough. Each time, she was diagnosed either with bronchiolitis or wheezy bronchitis and treated with bronchodilators, inhaled corticosteroids and antibiotics. However the symptoms recurred frequently, therefore further diagnostic approaches were initiated including chest-radiography, allergy tests (specific IgE) and otorhinolaryngeal examination, which revealed no abnormalities. At the age of two years the girl was admitted to our hospital because of an acute exacerbation of her pulmonary situation. She presented in reduced physical condition with respiratory distress at rest, wheezing and dry cough. Inhalation therapy with beta agonists showed no success, there were no signs of a severe viral or bacterial infection. Based on her medical history of several episodes with recurrent, therapy-refractory wheezing without signs of an infection and her acute condition of severe respiratory distress we decided to extend the diagnostic approach and underwent flexible bronchoscopy under general anesthesia. The larynx appeared with a mild malacia, the branching of the right upper lobe bronchus (RUL) was atypical in the anterior aspect of the carina followed by tracheal stenosis. The main bronchi could not be entered with a two millimeter bronchoscope (Figure 1). Therefore and to identify the exact anatomical situation a CT scan of the lung was performed with a three-dimensional reconstruction of the tracheo-bronchial system, which demonstrated a voluminous right upper lobe bronchus branching of the trachea at the level of the aortic arch, followed distally by a tracheal stenosis to the left (Figure 2). At a lower vertebral level than that of the normal carina (T5-6), the trachea was divided into two bronchi (bifurcatio tracheae), of which one proceeded to the left side of the lung (left main bronchus, LMB) and one adjacently with the pulmonary artery to the right (bridging bronchus, BB). This bridging bronchus upcoming from the left supplied the right lower and middle lobe. Whereas the right lower lobe was normally configured, the right middle lobe appeared hypoplastic. Parts of the ventral upper lobe were hyperinflated. Despite the small diameter of the left main bronchus, the left lung was normally ventilated. CT scan provided no indication of an associated vascular malformation e.g. a left sling pulmonary artery (SLPA). To exclude further anatomical variations of the cardiovascular, renal or gastrointestinal system echocardiography and abdominal ultrasound were performed and demonstrated no anomalies. 

Treatment with epinephrine inhalation and leucotriene receptor antagonist was initiated for several weeks and the patient was followed by our outpatient clinics every three months. Early intervention with physiotherapy, early use of mucolytic therapy and antibiotics were recommended during infections. During her three-years follow up period the girl has shown several croup-like attacks, but did well without further intervention or therapy.

## Conclusions

**Figure 3:**
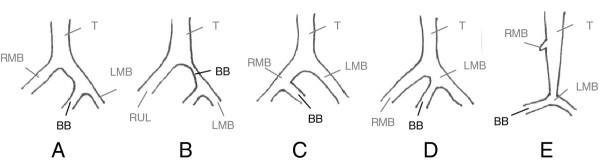
Different kinds of bridging bronchus published.

The bridging bronchus is a very rare anomaly, which was originally reported by Gonzalez-Crussi et al. in 1976 [4]. Until now, approximately 13 cases of this anomalous bronchial branching have been published worldwide [4-14]. A critical review of the pertinent literature showed, that the term “bridging bronchus” is defined differentially by several authors. As listed below there are 5 different interpretations of this term within these case reports (Figure 3A-E). 

The most common type is described in the original publication of Gonzalez-Crussi et al. [4], where the bridging bronchus was defined as a large bronchial branch which originated in the left main-stem bronchus, bridged the mediastinum from the ipsi- to the contralateral lung and provided the right lower and middle lobe (Figure 3A). The right upper lobe was supplied by the right main bronchus branching directly off the trachea at level of the carina. Four further authors reported on the same kind of bridging bronchus as an anatomic variation of the bronchial tree, where all patients apart from one died within the first six months [5-8]. Starshark et al. referred on a female newborn whose right lower lobe was supplied by a bronchus originating from the left main bronchus [5]. This newborn presented with several other congenital anomalies, i.e. imperforate anus, absent coccyx, horseshoes kidney and cardiovascular malformation. Medina and Lopez et al. described a male newborn with an imperforate anus at birth as well, a sling left pulmonary artery (SLPA) and a bridging bronchus as mentioned above [6]. As reported by Bertucci et al. the autopsy of a six month old female revealed an abnormal bronchus to the right lower lobe, descending from the left main bronchus associated with a SLPA [7]. Stokes and coworkers reported about a bridging bronchus similar to the one described by Gonzalez-Crussi, which was successfully treated by surgical reconstruction in the use of auricular cartilage from the patient’s ear (Figure 3A) [8]. Our patient had comparable bronchial malformation as the one reported by Gonzalez-Crussi et al. based on the definition that the bridging bronchus crossed the mediastinum from left to the right [4]. However there were no associated malformations of the cardiovascular, skeletal or gastrointestinal system as described by others.

Another type of bridging bronchus was reported by Topcu et al. and Grillo at al. as a stenotic bronchus between the branch of the right upper lobe bronchus and the carina formed by the bronchus supplying the right middle-lower lobe und the left main stem bronchus (Figure 3B) [9,15,16].

A third form of bridging bronchus is specified in the literature by Hawass et al. [10], who reported about a case of bridging bronchus, were the left lower lobe was provided by a bronchial branch that arose from the right main stem bronchus. This type is in accordance with the mirror image of the bridging bronchus in its original form named by Gonzalez-Crussi (Figure 3C) [4].

Rishavy et al. performed bronchoscopy in a five months old girl because of the suspicion of foreign body aspiration and revealed a fourth type of bridging bronchus [11]. Here a low carina presented with three orifices which the authors choose to denote from left to right: the left main stem bronchus (LMB), the bridging bronchus (taking of the LMB) and the right main stem bronchus (RMB). On the bronchogram the bridging bronchus appeared to supply the right lower lobe similar the original report by Gonzalez-Crussi et al. (Figure 3D) [4].

The fifth type of a bridging bronchus is associated with an abortive right main stem bronchus, represented by a small diverticulum of the trachea and an absent right upper lobe. There are two case reports about this form of bridging bronchus in the literature, where the trachea appeared abnormally long with a stenotic (“carrot-shaped”) caliber of the distal part [6,12]. The “pseudocarina”, localized at a lower thoracic vertebral level than for the normal position of the carina, where the bridging bronchus arises from the left main bronchus and supplies the right lung. Due to an increased bifurcation angle for the bridging bronchus and a normal angle for the left main bronchus, the “pseudocarina” appeared as an “inverted T”. In this type of malformation, the bridging bronchus can easily be taken for the right main bronchus (Figure 3E). In the report of Wells et al. further congenital anomalies of the cardiovascular system, i.e. double outlet right ventricle, pulmonary artery stenosis, SLPA and an absent gallbladder were presented [12].

Although different anatomic variations of bronchial branching are described in the literature, there are some identical clinical characteristics as well. Several authors reported about infants with bridging bronchus and a gestational weight of less than 2500 g [5,6,13]. With a weight of 2630 g our patient was small for gestational age as well. This could be caused by isolated bronchial branching anomalies or more likely by associated malformations of other organs.

Consistently the published reports showed that those patients with bridging bronchus and additional anomalies more often progress to cardiopulmonary failure than those without additional malformation. This fact seems to be extremely important for the prognosis. All 8 of the 13 reported patients with further congenital abnormalities - particularly of the cardiovascular system - died in infancy [4-7,12]. The reports about infants surviving the follow up period included three patients with isolated abnormalities of the tracheobronchial tree (without vascular anomalies), which were operated successfully [8,9,14]. One survivor suffered from an asymptomatic bridging bronchus, which was found incidental without the need of further intervention [11]. The study of Thiemann et al. in 1985 supports the data regarding the prognosis [17]. He and his coworkers investigated 2000 bronchograms within 136 cases of bronchial branching anomalies and found that isolated simple bronchial anomalies have no pathological value. Only branching abnormalities with malformations of the according blood vessels or lung tissue have the relevance of a real disease.

To the best of our knowledge this is the first report about a patient with a bridging bronchus in its “original form” without associated anomalies of the trachea-bronchial system or other organs. The pathogenesis of bronchial anomalies remains widely unknown and is controversially discussed in the literature. While some authors assume the origin in the embryonic lung development including the hypotheses of reduction, migration und selection, others postulate that the current embryonic theory could not account for bridging bronchus after formation of the main bronchial buds [5,18-21].

We found in our patient a bridging bronchus similar to Gonzalez-Crussi et al. [4] as a bronchial branch crossing the mediastinum from the ipsi- to the contralateral lung. However both, the definition of the carina used by Gonzalez-Crussi et al. and the anatomical location differ from our. While they defined the bifurcatio tracheae as the site where the right upper lobe bronchus branches directly of the trachea, the carina in our patient is located more distally and directed to the left side of the thorax due to a stenotic segment of the trachea (Figure 2). The concept of airway narrowing seems to be similar to the type of bridging bronchus reported by Topcu and Grillo et al. (Figure 3B) [9,15,16], but in contrast they defined the tracheal stenosis as the bridging bronchus. Berdon et al. published a classification of Wells and Landing with the trias of tracheobronchial narrowing, low carina and a pulmonary artery sling [22]. In summary bronchial branching malformation like bridging bronchus with or without stenosis is an important differential diagnosis in infants with respiratory distress under appropriate therapy. The case report demonstrates that further diagnostic approach should be established if children present with an atypical history of disease with recurrent wheezing episodes in absence of an infection and despite long-term therapy. While chest radiography fails to diagnose bronchial tree anomalies, bridging bronchus can be identified by bronchoscopy or CT scan. Further diagnostic approach may include MR angiography and abdominal sonography to evaluate additional malformations.

## Consent

Written informed consent was obtained from the patient’s parents for publication of this case report and any accompanying images. A copy of the written consent is available for review by the Series Editor of this journal.

## Abbreviations

BB: Bridging bronchus; RUL: Right upper lobe bronchus; LMB: Left main stem bronchus; RMB: Right main stem bronchus; BiT: Bifurcatio tracheae; T: Trachea; TS: Tracheal stenosis.

## Competing interests

The authors declare that they have no competing interests.

## Authors’ contributions

AS drafted the manuscript. KG performed the radiological diagnostics. CV performed the endoscopy and drafted the manuscript. All authors read and approved the final manuscript.

## Pre-publication history

The pre-publication history for this paper can be accessed here:

http://www.biomedcentral.com/1471-2431/12/110/prepub
